# Astrocytes Stimulate Microglial Proliferation and M2 Polarization In Vitro through Crosstalk between Astrocytes and Microglia

**DOI:** 10.3390/ijms22168800

**Published:** 2021-08-16

**Authors:** Sumin Kim, Youngsook Son

**Affiliations:** 1 Department of Genetics and Biotechnology, College of Life Science and Graduate School of Biotechnology, Kyung Hee University, Yong In 17104, Korea; sumk@khu.ac.kr; 2 Kyung Hee Institute of Regenerative Medicine (KIRM), KHU Medical Science Research Institute, Kyung Hee University Medical Center, Seoul 02447, Korea

**Keywords:** microglia, M1/M2 polarization, astrocyte, microglia and astrocyte crosstalk

## Abstract

Microglia are resident immune cells of the central nervous system that act as brain-specific macrophages and are also known to regulate the innate immune functions of astrocytes through secretory molecules. This communication plays an important role in brain functions and homeostasis as well as in neuropathologic disease. In this study, we aimed to elucidate whether astrocytes and microglia could crosstalk to induce microglial polarization and proliferation, which can be further regulated under a microenvironment mimicking that of brain stroke. Microglia in a mixed glial culture showed increased survival and proliferation and were altered to M2 microglia; CD11b^−^GFAP^+^ astrocytes resulted in an approximately tenfold increase in microglial cell proliferation after the reconstitution of astrocytes. Furthermore, GM-CSF stimulated microglial proliferation approximately tenfold and induced them to become CCR7^+^ M1 microglia, which have a phenotype that could be suppressed by anti-inflammatory cytokines such as IL-4, IL-10, and substance P. In addition, the astrocytes in the microglial co-culture showed an A2 phenotype; they could be activated to A1 astrocytes by TNF-α and IFN-γ under the stroke-mimicking condition. Altogether, astrocytes in the mixed glial culture stimulated the proliferation of the microglia and M2 polarization, possibly through the acquisition of the A2 phenotype; both could be converted to M1 microglia and A1 astrocytes under the inflammatory stroke-mimicking environment. This study demonstrated that microglia and astrocytes could be polarized to M2 microglia and A2 astrocytes, respectively, through crosstalk in vitro and provides a system with which to explore how microglia and astrocytes may behave in the inflammatory disease milieu after in vivo transplantation.

## 1. Introduction

Glial cells are non-neuronal cells in the central and peripheral nervous systems. The glial cells in the central nervous system (CNS) include oligodendrocytes, astrocytes, ependymal cells, and microglia [1]. In this study, we focused on microglia and astrocytes. Microglia are resident immune cells of the CNS such as brain-specific macrophages. In a healthy brain, microglia show a ramified morphology in their resting state and patrol the brain to regulate brain homeostasis [2]. Microglia become activated after brain injury, either classically activated or alternatively activated, and are referred to as M1 and M2 types. The activated microglia exhibit a morphological change from a ramified shape to rounded or elongated spindle shape and migrate to the injury site [3]. At the injury site, two types of activated microglia play opposite roles. M1 microglia play a pro-inflammatory role under brain injury by releasing harmful cytokines, which induce neuronal death, while M2 microglia release anti-inflammatory cytokines, clean up the debris at the injury site by phagocytosis, and induce tissue repair by the secretion of neuroprotective growth factors [4,5].

In the case of stroke, M2 microglia/macrophages increase within five days after injury. However, after M2 microglia reach the peak, they decrease, while M1 microglia/macrophages show steady enhancement until 14 days after injury [6]. Although it is unclear whether an increase in M1 microglia means an increasing M1 polarization of resting microglia or the skewing of M2 microglia to M1 microglia, an increase in M2 polarization or inhibition of M1 skewing are key for impeding the aggravation of injury and inducing tissue repair. Since the microglial population in the brain is low, it is important to increase microglial proliferation and reversibly convert M1 and M2 phenotypes in vitro for adoptive cell therapy. There are many previous studies regarding the regulation of microglial M1/M2 polarization. Interleukin-4 (IL-4) and interleukin-10 (IL-10) are well-known cytokines that induce M2 microglia, and tumor necrosis factor-α (TNF-α) and interferon-γ (IFN-γ) are representative inducers of M1 microglia [4,7,8]. Substance P (SP) is a neurotransmitter composed of 11 amino acids regulating immune modulation [9]. In our previous study, the injection of SP into a rat spinal cord injury model increased the number of M2 microglia/macrophages at the injury site [10]. Moreover, bone marrow monocytes/macrophages can be activated and induced to proliferate by GM-CSF treatment, and induced to polarize into either of the M1 or M2 phenotypes by pro-inflammatory cytokines, such as TNF-α and IFN-γ or anti-inflammatory cytokines, such as IL-4/13 and IL-10, respectively, where SP, known as a neurotransmitter or neurohormone, has been identified as a novel anti-inflammatory cytokine [11]. Microglia and macrophages play similar roles at the tissue level and share many markers. However, SP’s effect on microglia is unclear.

Astrocytes are the most abundant cells in the brain, and they maintain CNS homeostasis, provide structural support, and regulate ion, nutrient, and gas concentrations [12,13]. Astrocytes become reactive astrocytes after injury. Reactive astrocytes act as a double-edged sword because they play a dual role in CNS disease. Reactive astrocytes enhance damage by releasing pro-inflammatory cytokines, forming a glial scar, or alleviating the inflammation, and boosting tissue regeneration [14,15]. Just like microglia, recently, reactive astrocytes were also distinguished as two different types: A1 astrocytes play a harmful role, while A2 astrocytes are neuroprotective [16,17]. Although the roles of A1/A2 astrocytes are well known, more research on the inducers of A1/A2 astrocytes and the makers of both reactive astrocytes is needed. Furthermore, the crosstalk between astrocytes and neurons and microglia and neurons has been vigorously studied [18,19], whereas the crosstalk between astrocytes and microglia has been less studied. During CNS disease, astrocytes are activated later than microglia. Thus, studies of the crosstalk between microglia and astrocytes focused on astrocytes; studies on the crosstalk between these two cells with a focus on microglia and the correlation of both A1/A2 astrocytes and microglia are needed.

In this study, we aimed to elucidate whether astrocytes and microglia could crosstalk, especially focusing on the microglial phenotypic change and microglial polarization induced by SP. We found that microglia co-cultured with mixed glial cells for 14 days were polarized to the M2 phenotype and that microglial proliferation increased. Moreover, to further demonstrate the key cells that induced these microglial changes among the mixed glial cells, we co-cultured microglia and astrocytes. The microglia–astrocyte co-culture showed more proliferating cells, as confirmed by a BrdU-incorporation assay. In addition, to increase microglial proliferation, we treated the microglia with various cytokines and demonstrated that GM-CSF increased the proliferation of microglia and induced M1 polarization. This polarization was inhibited by sequential treatment with SP, IL-4, or IL-10 after GM-CSF treatment. Furthermore, we demonstrated that the reactive astrocytes of the co-culture were skewed to the A2 type by confirming A1/A2 specific marker expression. In conclusion, crosstalk between microglia and astrocytes increased microglial proliferation and M2 polarization, and SP was able to suppress the M1 polarization of microglia.

## 2. Results

### 2.1. Astrocytes in Whole Brain Cell Isolates Stimulated Microglial Survival and Proliferation in the Mixed Culture In Vitro

**Figure 1:**
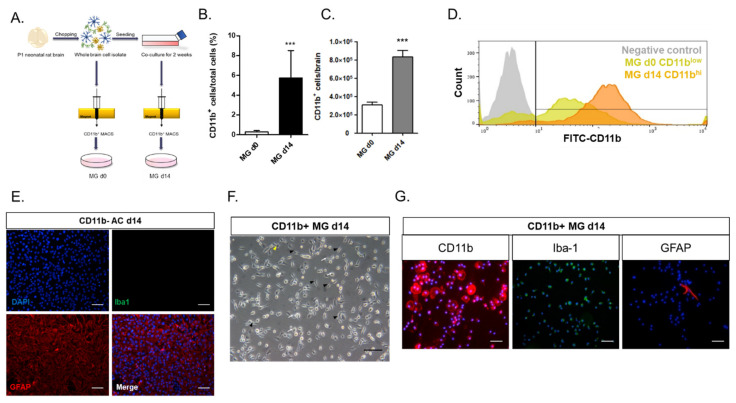
CD11b^−^/GFAP^+^ astrocyte from whole brain culture induced microglial proliferation and activation: (**A**) the experimental scheme of microglial isolation by MACS. Day 0 microglia were isolated immediately from whole brain cell isolates by CD11b+ MACS, and Day 14 microglia were isolated from the 2-week mixed glial cell culture; (**B**) the CD11b^+^ cell yield of MG d0 and MG d14 (*n* = 11); (**C**) the numbers of CD11b^+^ cells per brain for MG d0 and MG d14 (*n* = 11); (**D**) flow cytometry analysis of MG d0 and MG d14 with the microglia-specific marker CD11b. Fluorescent intensity of CD11b was enhanced in MG d14 compared to that of MG d0; (**E**) fluorescence images for CD11b^−^ cells sorted from the 2-week mixed glial cell culture stained for microglia-specific marker iba-1 and astrocyte-specific marker GFAP; (**F**) bright field image of MG d14. Yellow arrowhead: ramified microglia; black arrowhead: bipolar-shape microglia; white arrow: round-shape microglia; (**G**) fluorescence images of MG d14 stained with microglia-specific markers CD11b and iba-1 and astrocyte-specific marker GFAP. *** *p* < 0.001; scale bar = 100 μm.

To study the regulation of microglial phenotype by other cells in the brain in vitro, microglia were cultured immediately after the MACS isolation of whole brain cells with the microglial/macrophage-specific marker CD11b antibody, or CD11b^+^ microglia were isolated after two weeks of culture of whole brain cell isolates (Figure 1A). Only 0.3% of the total brain cell isolates were CD11b^+^ microglia immediately after the cell isolation (MG d0) (Figure 1B), most of which died approximately 5–7 days into the cell culture (data not shown). After two weeks of the culture of the whole brain cell isolates, the CD11b^+^ microglia increased to approximately 5.7% of the total cells (MG d14) (Figure 1B), similar to a previous report [20]. In the mixed culture of the whole brain cell isolates, the microglia increased by approximately three-fold from Day 0 to Day 14 (Figure 1C). Moreover, the level of CD11b expression increased low (CD11b^lo^) at Day 0 to high (CD11b^hi^) at Day 14, as shown by the FACS analysis (Figure 1D). 

In order to determine the cellular identity of approximately 94% of the other cells remaining in the two-week mixed culture of whole brain isolates, both CD11b^−^ and CD11b^+^ cells were collected and immunostained with antibodies against astrocyte or microglial markers (Figure 1E–G). Almost all the CD11b^−^ cells were iba-1-negative GFAP-expressing activated astrocytes. By contrast, most of the CD11b^+^ sorted cells at Day 14 showed bipolar and round morphologies of microglia in the activated state, but only some showed the ramified morphology of the resting microglia (Figure 1F). Approximately 99% of the CD11b^+^ cells were positive for the microglia-specific markers CD11b and Iba-1, and only 1% of the CD11b^+^ sorted cells were positive for the astrocyte-specific marker GFAP (Figure 1G). 

### 2.2. Astrocytes in the Mixed Culture Preferentially Induced M2-Type Microglia In Vitro

**Figure 2:**
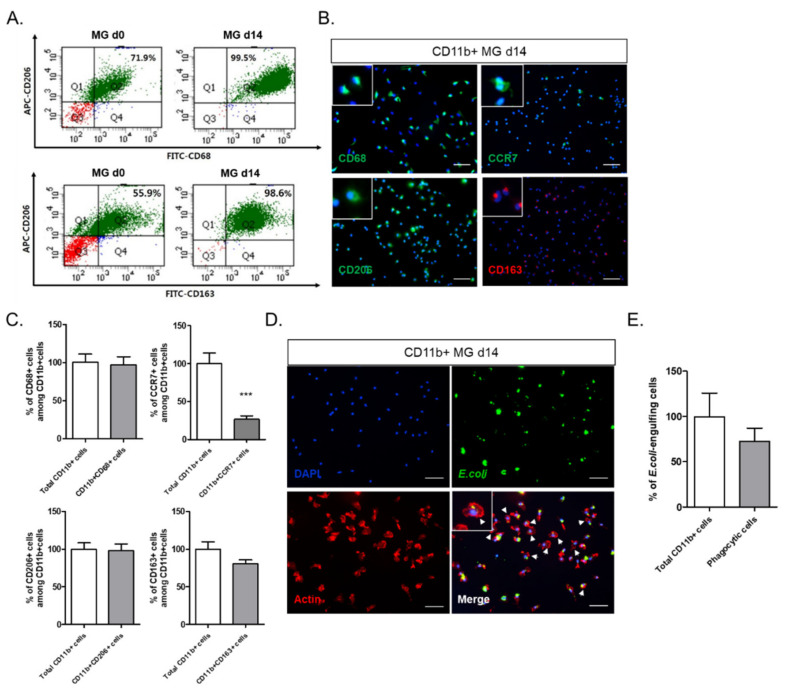
Phenotypic identity of MG d14 as M2 microglia: (**A**) flow cytometry analysis of MG d0 and MG d14 with CD206, CD68, and CD163 antibodies. CD206^+^CD68^+^ cells and CD206^+^CD163^+^ cells increased in MG d14; (**B**) fluorescence images of CD68, CCR7, CD206, and CD163 in MG d14. Left white box: magnified image of each marker positive cell. (**C**) quantitative analysis of CD68^+^, CCR7^+^, CD206^+^, and CD163^+^ cells among CD11b^+^ cells. (*n* = 5); (**D**) fluorescence image of the phagocytosis assay of MG d14. Left white box: magnified image of *E. coli*-particle-positive cells. White arrowhead: actin ruffles; (**E**) quantitative analysis of *E. coli*-particle-engulfing cells among MG d14 (*n* = 5). *** *p* < 0.001; scale bar = 100 μm.

The phenotypic change in the microglia over the two weeks of the ex vivo culture of the whole brain cell isolates was determined (Figure 2). CD11b^+^ sorted cells were analyzed by FACS using the antibodies CD68, as a marker of activated microglia, and CD206/CD163, as an M2 microglial marker (Figure 2A). CD68^+^CD206^+^ microglia increased from 71.9% at Day 0 to 99.5% at Day 14, and CD206^+^CD163^+^ microglia also increased from 55.9% at Day 0 to 98.6% at Day 14. To confirm the M2 polarization of microglia at Day 14, the microglia were stained with antibodies for CD68, CD206, and CD163, as activated M2 microglial markers, and CCR7, as an M1 microglial marker (Figure 2B). Almost all the microglia were positive for CD68 and CD206 expression, and approximately 80% ± 5.20% of the microglia were positive for CD163, whereas only 26.6% ± 4.52% of the CCR7^+^ M1 microglia were (Figure 2C). It seemed likely that the CCR7^+^ microglia overlapped with some of the CD68- and CD206-expressing microglia.

Since microglia are tissue-resident immune cells, they are expected to clean up cell debris or foreign particles by phagocytosis [21]. To examine the phagocytic function of microglia on Day 14 of culture, fluorescence-labeled *E. coli* particles were introduced to the microglial culture, and *E. coli*-engulfing microglia were visualized by actin cytoskeletal staining (Figure 2D). Many actively phagocytic microglia with actin ruffles were detected. Approximately 73% of the microglia were shown to have phagocytic function (Figure 2E). 

### 2.3. Reconstitution of Astrocytes in the Microglial Culture Enhanced Microglial Proliferation

**Figure 3:**
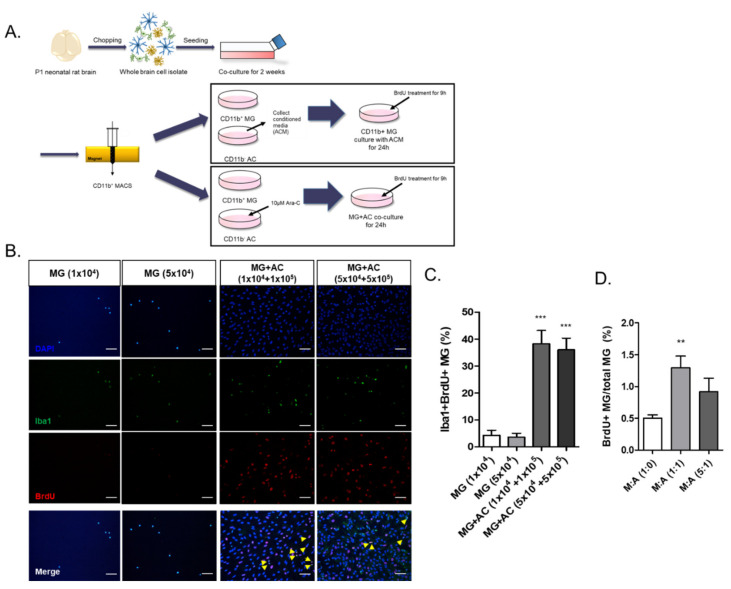
Astrocytes directly increased microglial proliferation: (**A**) the experimental scheme of microglial co-culture with astrocytes and treatment of ACM; (**B**) the BrdU-incorporation assay for the MG d14 co-cultured with astrocytes. Yellow arrowhead: Iba-1^+^BrdU^+^ cells; (**C**) the quantitative data of the percentage of BrdU^+^ cells under co-culture of astrocytes (*n* = 5); (**D**) quantitative data of the percentage of BrdU^+^ cells under treatment of ACM to MG d14 (*n* = 5). ** *p* < 0.01, *** *p* < 0.001, *n* = 3; scale bar = 100 μm.

Microglial survival and proliferation increased in the mixed culture of whole brain cell isolates, which were mainly GFAP^+^ activated astrocytes (Figure 1). We hypothesized that astrocytes might play an important positive role in microglial survival and proliferation by direct contact and/or by their secretomes. After CD11b^+^ cell sorting, a microglial culture was reconstituted with astrocytes or astrocyte-conditioned medium, and their effect on cell proliferation was measured by a BrdU-incorporation assay (Figure 3A). Since the initial ratio of CD11b^+^/CD11b^−^ cells in the 2-week mixed glial culture was approximately 1/15 (Figure 1B), CD11b^+^ microglia were re-plated at a 1:10 ratio of microglia:astrocytes pretreated with Cytarabine (Ara-C) or without astrocytes, and the Iba1^+^ BrdU-incorporating cells, representing proliferating microglia, were counted (Figure 3B,C). The BrdU^+^Iba-1^+^ microglia were almost ten times more numerous in the astrocyte co-culture than in the microglial single culture, which was observed at different cell densities. 

To examine the effect of the astrocyte secretome on microglial proliferation, astrocyte conditioned medium (ACM) was used to treat the microglial culture at a ratio of 1:1 or 1:5 with microglial culture medium (MCM), and a BrdU-incorporation assay was performed (Figure 3D). The BrdU-incorporating microglia were 2.5-fold more numerous at a 1:1 ratio of ACM:MCM than with MCM only; they were lower at a 1:5 ratio of ACM:MCM than at a 1:1 ratio.

### 2.4. Cytokine Profiles of Microglia, Astrocytes, and Their Co-Culture 

**Figure 4:**
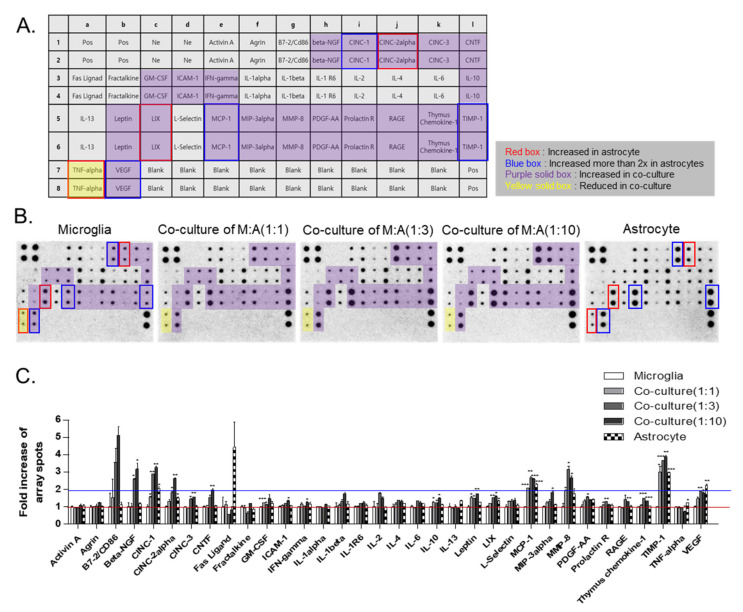
The secretome analysis of single or co-culture of microglia and astrocytes with rat cytokine array: (**A**) cytokine array map. The array can detect 34 soluble mediators. Pos: positive control; Ne: negative control. (**B**) The cytokine arrays were performed with conditioned medium of microglia, astrocyte single culture, or microglia–astrocyte co-culture in various ratios. (**C**) The quantitative data of the fold increase in array spots between microglia, astrocyte single culture, and microglia–astrocyte co-culture. Red line: 1x baseline of array spots; Blue line: 2x baseline of array spots. * *p* < 0.05, ** *p* < 0.01, and *** *p* < 0.001.

In order to explore the difference in the profiles of the cytokines secreted from astrocytes and/or microglia, conditioned media for microglia only, astrocytes only, and an astrocyte/microglia co-culture at different cell ratios were collected for one day and analyzed by using a rat cytokine array (Figure 4A–C). Most of the cytokines, activin A, agrin, β-NGF, CINC-3, CNTF, fractalkine, GM-CSF, ICAM-1, IFN-γ, IL-1α, IL-1β, IL-1R6, IL-2, IL-4, IL-6, IL-10, IL-13, leptin, L-selectin, MIP-3α, MMP-8, PDGF-AA, prolactin R, RAGE, and thymus chemokine, were similarly expressed in both the microglia and astrocytes. However, some cytokines, such as CINC-1 (CXCL-1, a neutrophil chemoattractant), CINC-2α, LIX, TNF−α, MCP-1 (CCL2 chemokines), TIMP-1, and VEGF were expressed more highly in astrocytes than microglia. Among them, CINC-1, MCP-1, TIMP-1, and VEGF were expressed two times more highly in astrocytes than in microglia. Other cytokines, such as β-NGF, CINC-1, CINC-2α, CINC-3, CNTF, GM-CSF, ICAM-1, IFN-γ, IL-10, leptin, LIX, MCP-1, MIP-3α, MMP8, PDGF-AA, prolactin R, RAGE, thymus chemokine-1, TIMP-1, and VEGF, were elevated in the microglia co-cultured with astrocytes, which are known to be involved in inflammation, neuronal migration and proliferation, and vascular repair and remodeling. Among them, only TNF-α was reduced in the co-culture. 

### 2.5. TNF-α and IFN-γ Further Activated M2 Microglia to iNOS- and IL-1β−Expressing M1 Microglia, Which Was Partially Inhibited by SP

**Figure 5:**
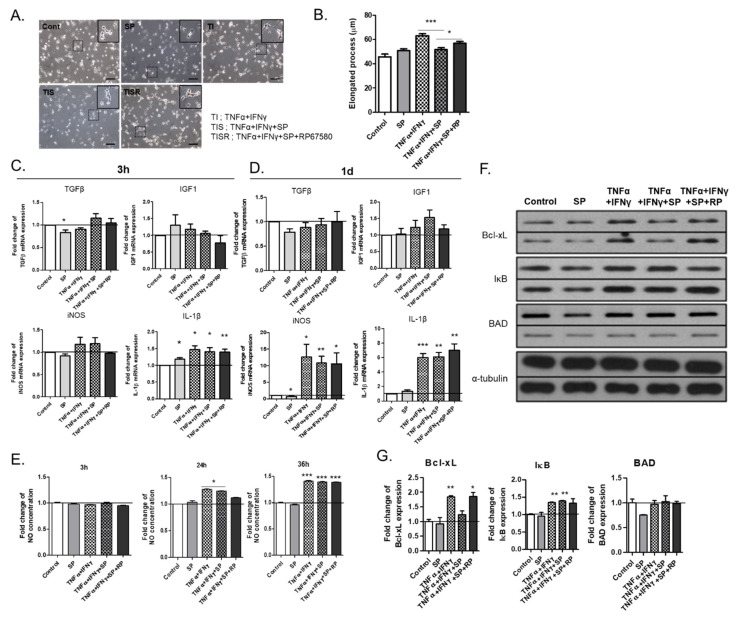
M1 polarization of MG d14 induced by treatment with TNF-α and IFN-γ: (**A**) Bright field images of microglia cultured with TNF-α and IFN-γ; microglia cultured with TNF-α, IFN-γ, and SP; and microglia cultured with TNF-α, IFN-γ, SP, and RP67580 (RP, an NK1 receptor antagonist). (**B**) quantitative data for elongated processes of microglia under various cytokine treatments; (**C**,**D**) quantitative data for mRNA expression of TGF-β, IGF1, iNOS, and IL-1β at 3 h after treatment (**C**) and one day after treatment (**D**) (*n* = 3). Black line: 1x baseline of mRNA expression; (**E**) Quantitative data of NO production in various cytokine combinations at different time points (*n* = 3). Black line: 1x baseline of NO concentration; (**F**) the Western blot analysis of Bcl-xL, IκB, and BAD in microglia cultured with TNF-α, IFN-γ, SP, and RP67580; (**G**) quantitative data of Bcl-xL, IκB, and BAD expression (*n* = 3). Black line: 1x baseline of protein expression. * *p* < 0.05, ** *p* < 0.01, and *** *p* < 0.001; scale bar = 100 μm.

It was explored whether M2 microglia in the mixed glia culture could be further converted to the M1 phenotype upon exposure to pro-inflammatory cytokines, such as TNF-α and IFN-γ, which can also be modulated by the co-existence of the neuropeptide SP, also known as M2 cytokine [11]. TNF-α and IFN-γ altered the morphology of primary microglia from a round amoeboid and short bipolar morphology to the bipolar morphology with an elongated process (Figure 5A,B). SP itself did not significantly alter the morphological change but partially inhibited the TNF-α- and IFN-γ-mediated process elongation, which was nullified by co-treatment with the NK-1 receptor antagonist RP67580. To define the phenotype of TNF-α- and IFN-γ-activated microglia, qPCRs of TGF-β and IGF1 for M2 microglia and iNOS and IL-1β for Μ1 microglia were performed (Figure 5C,D). TNF-α and IFN-γ did not affect the expression of TGF-β and IGF1 significantly but markedly increased the expression of iNOS and IL-1β at one day post-treatment. Along with iNOS induction, elevated NO production was observed in TNF-α- and IFN-γ-treated microglia (Figure 5E). Accordingly, TNF-α- and IFN-γ-activated microglia exhibited an M1 phenotype. Furthermore, TNF-α and IFN-γ increased Bcl-xL expression, which was inhibited by SP co-treatment (Figure 5F,G). The microglia in the mixed glial culture skewed the M2 phenotype, which could be further polarized to the M1 phenotype upon the exposure to the pro-inflammatory cytokines TNF-α and IFN-γ.

### 2.6. GM-CSF Stimulated Microglial Proliferation and CCR7^+^ M1 Phenotype In Vitro, Which Was Subsequently Suppressed by Anti-Inflammatory Cytokines Such as IL-4, IL-10, and SP 

**Figure 6:**
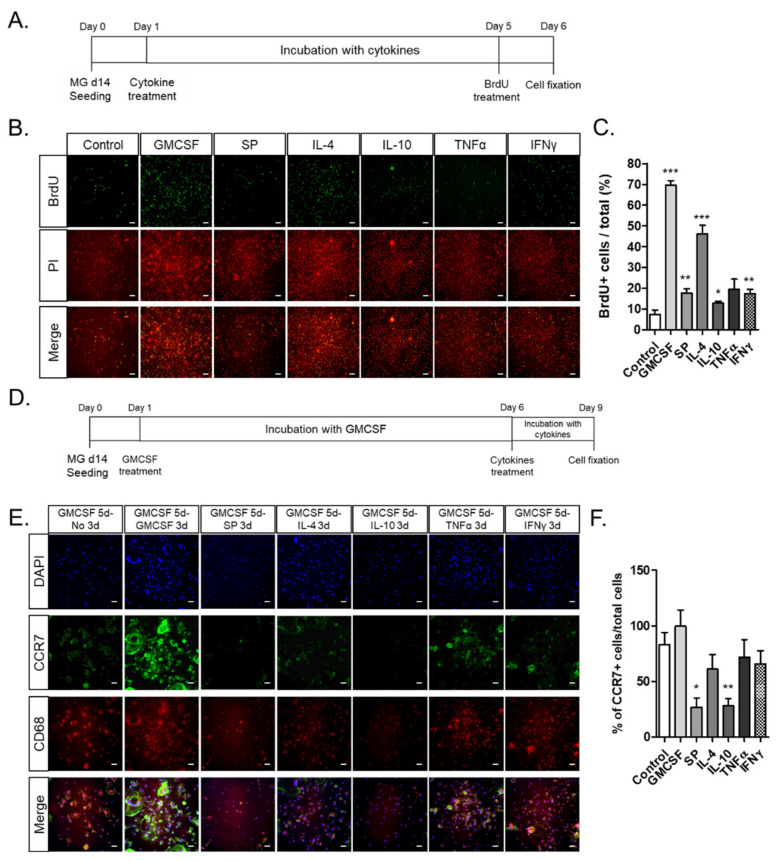
Activation of MG d14 cell proliferation and M1 skewing by GM-CSF and suppression of M1 polarization by GM-CSF removal and subsequent treatment with M2 cytokines: SP, IL-4, and IL-10. (**A**–**C**) the experimental scheme of the cytokine and BrdU treatment. MG d14 were incubated with the following cytokines for five days: GM-CSF, SP, IL-4, IL-10, and TNF-α or IFN-γ (**A**). The BrdU-incorporation assay (**B**) and the quantitative data for the percentage of BrdU^+^ cells (**C**); (**D**–**F**) the experimental scheme of the cytokine and BrdU treatment. MG d14 were pre-treated with GM-SCF for five days and then incubated with the following cytokines for three days: GMCSF, SP, IL-4, IL-10, and TNF-α or IFN-γ (**D**); fluorescence images stained with activated microglia marker CD68 and M1 microglia marker CCR7 (**E**). Quantitative data for CCR7^+^ cells among total cells (**F**) (*n* = 5); * *p* < 0.05, ** *p* < 0.01, and *** *p* < 0.001; scale bar = 100 μm.

To explore whether GM-CSF could stimulate microglial proliferation as well as phenotypic conversion, microglia with the M2 phenotype were incubated with different cytokines, and the BrdU-incorporating cells were counted (Figure 6A–F). GM-CSF and IL-4 stimulated microglial proliferation approximately ten-fold and six-fold, respectively, compared to that of the non-treated microglia, even though only marginal stimulation was observed in the SP, TNF-α, and IFN-γ treatment (Figure 6A–C). Then, it was explored whether GM-CSF-treated microglia could be further converted to M1/M2 phenotypes upon treatment with anti-inflammatory cytokines or pro-inflammatory cytokines (Figure 6D–F). Most of the microglia treated with GM-CSF showed high expression of the M1 microglial marker CCR7 and activated microglial marker CD68, which was generally maintained by subsequent TNF-α and IFN-γ treatment, but cell proliferation was inhibited rather than sustained by the GM-CSF treatment. However, the CCR7 expression in GM-CSF-treated microglia was markedly reduced by the subsequent removal of GM-CSF and treatment with anti-inflammatory cytokines such as IL-4, IL-10, and SP. Thus, microglia can be markedly expanded by GM-CSF treatment, and the M1 microglial phenotype can be repressed by subsequent treatment with anti-inflammatory cytokines.

### 2.7. Reactive Astrocytes in the Co-Culture Were Skewed to A2 Astrocytes, Which Could Be Induced to A1 Astrocytes upon TNF-α and IFN-γ Treatment

**Figure 7:**
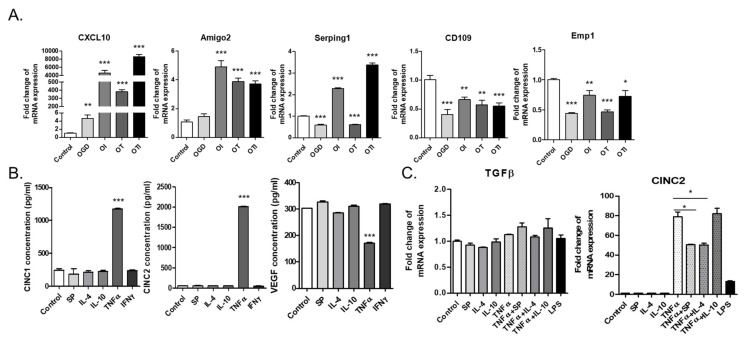
Phenotypic identity of reactive astrocytes in the mixed glial cell culture as A2 astrocytes: (**A**) quantitative analysis of AC d14 with CXCL10 (pan reactive astrocyte marker), Amigo2, Serping1 (A1 astrocyte markers), CD109, and Emp1 (A2 astrocyte markers) mRNA expression. (*n* = 3) OGD: oxygen glucose deprivation; OI: IFN-γ treatment after OGD; OT: TNF-α treatment after OGD; OTI: TNF-α + IFN-γ treatment after OGD. (**B**) ELISA of CINC-1, CINC-2, and VEGF secreted by AC d14 cultured with various cytokines (*n* = 3); (**C**) quantitative analysis of TGF-β and CINC-2 mRNA expression in AC d14 cultured with various cytokines (*n* = 3). * *p* < 0.05, ** *p* < 0.01, and *** *p* < 0.001.

In order to examine the phenotypic alteration of astrocytes in the co-culture with microglia, GFAP^+^ astrocytes were exposed to oxygen glucose deprivation (OGD) conditions, similar to the stroke microenvironment, in combination with the inflammatory cytokines TNF-α and IFN-γ (Figure 7A). Based on qPCR analysis, OGD did increase the expression of CXCL10, a neutrophil chemoattractant, which was markedly elevated by TNF-α and IFN-γ exposure, approximately 400- and 6000-fold, respectively. In addition, TNF-α and IFN-γ markedly elevated the expression of A1 astrocyte markers, such as Amigo 2 and Serping 1, where IFN-γ seemed to work more potently as an A1 inducer than the known A1 inducer TNF-α. By contrast, A2 astrocyte markers, such as CD109 and Emp1, were reduced by TNF-α and IFN-γ treatment.

Then, the modulation of A1/A2 phenotype by pro-inflammatory or anti-inflammatory cytokines was determined (Figure 7B,C). TNF-α markedly increased CINC-1 and CINC-2, which are major cytokines in astrocytes, but not VEGF, based on an ELISA and CINC-2 gene expression analysis via real-time PCR analysis. Anti-inflammatory cytokines, such as IL-4, IL-10, and SP, did not affect CINC 1/2 expression, and none of the cytokines affected TGF-β expression.

## 3. Discussion

In this study, we investigated the crosstalk between microglia and astrocytes and their phenotypic conversion in vitro. Over two weeks of culture, microglia isolated from neonatal rat brains by CD11b^+^ MACS isolation showed an increase in cell number (Figure 1), cells positive for M2 microglial markers, and phagocytotic microglia (Figure 2). This supports the concept that microglia derived from the neonatal rat brain can proliferate in a mixed culture of whole brain isolates and preferentially transform to M2-type microglia with phagocytic function. In addition, we characterized the non-microglial cell population in the mixed glial cell culture by staining with microglia- and astrocyte-specific markers. Almost 99% of the CD11b^−^ cells were found to be GFAP-positive astrocytes (Figure 1E). Thus, astrocytes are main cellular components in mixed cultures of whole brain cell isolates, which may provide a favorable microenvironment for microglial survival and proliferation. Moreover, the stimulation of astrocytes was demonstrated with the reconstitution of astrocytes in the CD11b^+^ MACS-sorted microglial culture and enhanced by astrocyte conditioned medium (Figure 3B–D). However, microglial cell proliferation was significantly higher in direct cell–cell contact in the microglia–astrocyte co-culture than in the conditioned medium. Therefore, astrocytes clearly secrete some factors stimulating microglial proliferation, but direct cellular contact with astrocytes further stimulates microglial proliferation. Additionally, cytokine arrays for microglial culture, astrocyte culture, and microglial and astrocyte co-culture in different cell ratios showed that both cells secrete many cytokines involved in immune responses, neurotrophic responses, tissue repair, and tissue remodeling (Figure 4), which may work in coordination in influencing microglial proliferation as well as M2 polarization. In particular, astrocytes in the mixed glial culture also seemed to acquire the A2 phenotype. Finally, it was shown that M2 microglia and A2 astrocytes could be further converted to M1 microglia and A1 astrocytes under an inflammatory microenvironment and stroke-mimicking environment, and TNF-α and IFN-γ increased Bcl-xL expression of microglia, suggesting self-defense mechanism from NO-induced apoptosis [22] (Figure 5, Figure 6 and Figure 7). In addition, astrocytes in microglia co-culture could be very susceptibly induced to A1 astrocyte phenotype by IFN-γ or TNF-α and CINC-1/2 expression could be applied as a whole marker for TNF-α -inducible A1 astrocyte. Thus, this study demonstrated crosstalk between microglia and astrocytes facilitating M2 microglia and A2 astrocytes, which can be further regulated under a variety of cytokine microenvironments, influencing their behavior in the in vivo environment after transplantation as adoptive cell therapy.

In addition, M2 microglia are not terminally differentiated but can proliferate and polarize to M1 microglia upon exposure to astrocyte-secreted cytokines, such as GM-CSF; M1 microglia have a phenotype that can also be further modulated by anti-inflammatory cytokines. Therefore, M1/M2 polarization may be specifically interpreted in the context of the cytokine milieu in the vicinity. In the brain microenvironment after ischemic injury, many inflammatory cytokines can activate M1 microglial polarization [23,24]. As shown by our data, M2 microglia can be polarized to M1 microglia upon exposure to inflammatory cytokines such as TNF-α and IFN-γ (Figure 5). M2 microglia play an anti-inflammatory role in CNS injury by the secretion of many growth factors and cytokines that promote tissue repair. However, applying M2 microglia as a cell therapy is quite challenging because a microglial cell source, such as the brain, is difficult to isolate from humans, the number of microglia in the brain is too low, and the injury environment can polarize the injected M2 microglia to M1 microglia. Thus, for adoptive cell therapy with M2 microglia to treat a variety of neurodegenerative diseases that have chronic inflammatory backgrounds, it may be technically important to increase M2 microglial cell populations by in vitro astrocyte co-culture, GM-CSF priming, and the subsequent induction of M2 polarization with IL-4, IL-10, and SP. However, the therapeutic effect of injecting M2 microglia to the stroke model and the maintenance of their phenotypes must be further demonstrated. In addition, SP shows an immunosuppressive role in both microglia and astrocytes, but further research is still needed to determine whether SP can skew M1 microglia to M2 microglia in conjunction with different cytokines and if SP can be a novel inducer of A2 astrocytes.

In a previous study, the neuroprotective crosstalk between astrocytes and microglia modulated TGF-β and IL-10 under an LPS-induced injury environment [25]. According to this study, the astrocytes in the microglial co-culture were most likely skewed to A2 astrocytes, and IL-10 secretion was increased in the co-culture condition, demonstrating the neuroprotective crosstalk between astrocytes and microglia under normal status. These cultured A2 astrocytes could be induced to the A1 phenotype by TNF-α and IFN-γ in the OGD condition; IFN-γ may be the most potent A1 inducer in the stroke environment (Figure 7). Moreover, CINC-1 and CINC-2, which were shown to be major cytokines released from astrocytes based on a cytokine array (Figure 4), may be considered to be novel markers of A1 astrocytes, since TNF-α, a known A1-inducing cytokine, strongly stimulated CINC-1/2 secretion and expression (Figure 7B). Therefore, this microglia–astrocyte co-culture system can be further applied to elucidate the phenotypic conversion of the A1/A2 astrocytes and the bidirectional communication between microglia and A1 and A2 reactive astrocytes in a variety of neuropathology-mimicking microenvironments.

In many previous studies, the roles of the Th 1 and Th 2 cytokines were well investigated under many neurodegenerative and autoimmune diseases. Th 1 cytokines, such as TNF-α and IFN-γ, are known to lead the progression of autoimmune diseases such as type 1 diabetes and multiple sclerosis (MS) [26,27,28], while IL-4, IL-10, and IL-13 refer to Th 2 cytokines and inhibit the pro-inflammatory response and reduce damage [29,30,31]. Furthermore, IL-37 plays an anti-inflammatory role and suppresses the pathogenesis of MS [32,33]. Moreover, the macrophage migration inhibitory factor (MIF) is a pleiotropic cytokine produced by epithelial cells, immune cells, astrocytes, and neurons. In neurodegenerative conditions, such as Alzheimer’s disease (AD), Parkinson’s disease (PD), and amyotrophic lateral sclerosis (ALS), MIF plays a dichotomic role. In PD and ALS, MIF inhibits the apoptosis of neuronal cells via suppressing inflammation by reducing the number of microglia/macrophages, which can result in neuroprotection [34]. However, in the case of AD, MIF induces pathogenic progression [35]. These cytokines may affect the microglial and astrocyte polarization under the injury environment, causing inflammation or suppression. The in vitro co-culture system in this study can be a good tool with which to develop expectations for how microglia and astrocytes may behave in the disease milieu after in vivo transplantation under brain injury and how these cells directly crosstalk and regulate the inflammatory responses to various cytokine stimuli.

## 4. Materials and Methods

### 4.1. Animals

All the animal experiments were approved by the Animal Studies Committee of Kyung Hee University (KHUASP #16-012 approved on 1 July 2016) in Yong In, Korea, and performed under the Institutional Animal Care and Use Committee (IACUC) guidelines.

### 4.2. Mixed Glial Cell Culture and Isolation of Microglia

The mixed glial cell culture was prepared from the brain of postnatal day (P) 1–3 inbred Lewis rats (Orientbio, Sungnam, Korea), modified as previously described [36]. Briefly, the meninges and the blood vessels of the whole brain were removed under a dissecting microscope. Then, the brain was minced into small pieces in serum-free Dulbecco’s modified Eagle’s medium/nutrient mixture F12 mixture (1:1) (DMEM/F12, Gibco, Grand Island, NY, USA) and centrifuged. The pellet was re-suspended in DPBS (Welgene, Daegu, Korea). After washing, the cells were seeded in 75 cm^2^ culture flasks in 15 mL of DMEM/F12 (1:1) containing 10% FBS (Gibco, Grand Island, NY, USA), 2 mM L-glutamine (Welgene, Daegu, Korea), 50 U/mL penicillin, and 50 mg/mL streptomycin (Welgene, Daegu, Korea). The cultures were maintained at 37 °C in a humidified atmosphere of 5% CO_2_ and 95% air. The medium was changed every three days, and the culture was maintained for 14 days.

To harvest microglia, microglia were isolated from the mixed glial cell culture on Day 14 using magnetic-activated cell sorting (MACS). All the glial cells were detached using 0.25% trypsin–EDTA, which was then neutralized with complete culture medium. Then, the cells were washed with PBS and re-suspended to a cell density of 1 × 10^7^ cells/mL. The cells were incubated with a CD11b antibody (1:100, BD Bioscience, San Jose, CA, USA) for 5 min at 4 °C and anti-mouse IgG microbeads (1:5, Miltenyi, Bergisch Gladbach, Germany) for 15 min at 4 °C. The cells were washed twice with PBS. During washing, the MACS column was equilibrated by applying 10 mL of autoMACS rinsing solution (Miltenyi, Bergisch Gladbach, Germany). The cell suspension was applied to the LS column (Miltenyi, Bergisch Gladbach, Germany) and allowed to pass through the resin by gravity flow. The column was washed twice with autoMACS rinsing solution. Then, the column was taken off from the magnet, and the CD11b^+^ microglia were eluted. The microglia were seeded into 6-well plates at a density of 1 × 10^5^ cells/mL or seeded into 24-well plates at a density of 5 × 10^4^ cells/mL and incubated with DMEM/F12 (1:1) supplemented with 10% FBS (Gibco, Grand Island, NY, USA), 2 mM L-glutamine (Welgene, Daegu, Korea), 50 U/mL penicillin, and 50 mg/mL streptomycin (Welgene, Daegu, Korea) at 37 °C in a humidified atmosphere of 5% CO_2_ and 95% air.

### 4.3. Phenotype Conversion of M1 and M2 Microglia In Vitro

To investigate the phenotypical changes of microglia, MG d14 were pre-treated with 10 ng/mL GMCSF (R&D Systems, Minneapolis, MN, USA) for 5 days and then incubated with the following cytokines for 3 days: 10 ng/mL GMCSF (R&D systems, Minneapolis, MN, USA), 100 nM Substance P (SP, Sigma, St. Louis, MO, USA), 10 ng/mL rat IL-4 (R&D systems, Minneapolis, MN, USA), 10 ng/mL rat IL-10 (R&D systems, Minneapolis, MN, USA), 10 ng/mL TNF-α (Sigma, St. Louis, MO, USA), and 20 ng/mL IFN-γ (R&D systems, Minneapolis, MN, USA).

### 4.4. Astrocyte Culture and OGD In Vitro

CD11b^−^ astrocytes were collected by CD11b MACS. The cells were incubated with DMEM/F12 (1:1) supplemented with 10% FBS (Gibco, Grand Island, NY, USA), 2 mM L-glutamine (Welgene, Daegu, Korea), 50 U/mL penicillin, and 50 mg/mL streptomycin (Welgene, Daegu, Korea) at 37 °C in a humidified atmosphere of 5% CO_2_ and 95% air. The culture was maintained for 14 days.

To mimic the in vivo stroke environment, CD11b^−^ astrocytes isolated by CD11b MACS were seeded into 6-well plates at a density of 1 × 10^6^ cells/mL and incubated with no-glucose DMEM (Gibco, Grand Island, NY, USA) at 37 °C in a humidified atmosphere of 1% O_2_ and 5% CO_2_ air for 24 h using a Galaxy 48R incubator (Eppendorf, Hamburg, Germany). Then, the cells were treated with 10 ng/mL TNF-α (Sigma, St. Louis, MO, USA) and 10 ng/mL IFN-γ (R&D systems, Minneapolis, MN, USA) for 4 h at 37 °C in a humidified atmosphere of 5% CO_2_ and 95% air, mimicking the reperfusion following ischemic stroke. 

### 4.5. Phagocytosis Assay for Microglia

On Day 1, the MG d14 were incubated with 25 μg/mL *E. coli* BioParticle (Thermo, Waltham, MA, USA) for 30 min and fixed with 3.7% formaldehyde. The cells were permeabilized with 0.2% Triton X-100 for 5 min and incubated with TRITC-conjugated phalloidin (Sigma, St. Louis, MO, USA, 1:1000) for 30 min at RT. Then, the cells were mounted with Vectashield mounting medium containing DAPI (Vector, Burlingame, CA, USA), and immunofluorescence images were obtained using the Leica DMI 4000 B fluorescence microscope (Leica Microsystems, Wetzlar, Germany).

### 4.6. Reconstitution of Astrocyte Feeder on Microglial Culture

Astrocytes were cultured as described above. When the cells reached confluence, 10 μM Ara-C (Sigma, St. Louis, MO, USA) was added for 4 days, and the cells were washed with PBS. Then, the MG d14 isolated from the mixed glial cell culture was added on the top of the astrocyte feeder and incubated for 24 h in DMEM/F12 (1:1) supplemented with 10% FBS (Gibco, Grand Island, NY, USA), 2 mM L-glutamine (Welgene, Daegu, Korea), 50 U/mL penicillin, and 50 mg/mL streptomycin (Welgene, Daegu, Korea) at 37 °C in a humidified atmosphere of 5% CO_2_ and 95% air.

### 4.7. Flow Cytometry

To harvest MG d0, the brains of P1–3 inbred Lewis rats (Orientbio, Sungnam, Korea) were minced and the CD11b^+^ cells isolated using MACS without a mixed glial cell culture. The MG d14 were isolated from the mixed glial cell culture and trypsinized. Both groups of cells were blocked with 1% bovine serum albumin (BSA, Sigma, St. Louis, MO, USA) and incubated with primary antibody for 30 min at 4 °C: CD11b (BD Bioscience, San Jose, CA, USA, 1:100), CD206 (Abcam, Cambridge, MA, USA, 1:200), CD163 (AbD Serotec, Oxford, UK, 1:100), and CD68 (Millipore, Burlington, MA, USA, 1:200). After washing the cells with 0.1% BSA solution, the cells were incubated with secondary antibody for 30 min at 4 °C: APC donkey anti-rabbit (Jackson ImmunoResearch, W Baltimore Pike, PA, USA, 1:500) and FITC goat anti-mouse (Abcam, Cambridge, MA, USA, 1:500). The data were acquired with the BD FACSDiva 7.0 software.

### 4.8. Immunofluorescence Staining

The microglia and astrocytes were fixed with 3.7% formaldehyde (Sigma, St. Louis, MO, USA) and permeabilized by treatment with 0.2% Triton X-100 in PBS for 5 min. Then, the samples were blocked using 20% normal goat serum (Vector, Burlingame, CA, USA) for 1 h at RT. For immunofluorescence staining, the samples were incubated with primary antibodies at 4 °C overnight: CD11b (BD Bioscience, San Jose, CA, USA, 1:100), ionized calcium-binding adapter molecule-1 (Iba-1, Sigma, St. Louis, MO, USA, 1:200), CD163 (AbD Serotec, Oxford, UK, 1:100), CD206 (Abcam, Cambridge, MA, USA, 1:200), CD68 (Millipore, Burlington, MA, USA, 1:500), CCR7 (Abcam, Cambridge, MA, USA, 1:500), and GFAP (Abcam, Cambridge, MA, USA, 1;500). The samples were then incubated with secondary antibodies for 1 h at RT: Alexa 488 goat anti-mouse (Invitrogen, Waltham, MA, USA, 1:1000), Alexa 488 goat anti-rabbit (Jackson ImmunoResearch, W Baltimore Pike, PA, USA, 1:1000), Cy3 goat anti-mouse (Jackson ImmunoResearch, W Baltimore Pike, PA, USA, 1:1000), and Cy3 goat anti-rabbit (Jackson ImmunoResearch, W Baltimore Pike, PA, USA, 1:1000). The cells were mounted with Vectashield mounting medium containing DAPI (Vector, Burlingame, CA, USA), and immunofluorescence images were obtained using a Leica DMI 4000 B fluorescence microscope (Leica Microsystems, Wetzlar, Germany). The cell counting was performed at 200x magnification using Adobe Photoshop CS6 software.

### 4.9. BrdU-Incorporation Assay

The ACM was collected from CD11b^−^ astrocyte culture and mixed with fresh MCM at two different ratios (i.e., 1:1 and 1:5). The diluted conditioned medium was used to treat microglia, which were incubated for 24 h at 15 h after seeding; then, 20 μM BrdU (Sigma, St. Louis, MO, USA) was added to the culture medium, and the cells were incubated for 9 h. The microglia were fixed with ice-cold 100% absolute methanol (Merck, Darmstadt, Germany) for 5 min. Then, the microglia were incubated with anti-BrdU antibody (Roche, Basel, Schweiz, 1:25) overnight at 4 °C and with Alexa 488 goat anti-mouse antibody (Invitrogen, Waltham, MA, USA, 1:1000) for 1 h at RT. The nucleus was stained with propidium iodide (PI) (Sigma, St. Louis, MO, USA, 1:1000) for 30 min at RT. The sample was mounted with Vectashield mounting medium (Vector, Burlingame, CA, USA), and immunofluorescence images were obtained using the Leica DMI 4000 B fluorescence microscope (Leica Microsystems, Wetzlar, Germany). The BrdU-positive cells were counted at 100x magnification using Adobe Photoshop CS6 software.

The MG d14 were incubated with the following cytokines for 5 days: 10 ng/mL GMCSF (R&D systems, Minneapolis, MN, USA), 100 nM SP (Sigma, St. Louis, MO, USA), 10 ng/mL rat IL-4 (R&D systems, Minneapolis, MN, USA), 10 ng/mL rat IL-10 (R&D systems, Minneapolis, MN, USA), 10 ng/mL TNF-α (Sigma, St. Louis, MO, USA), and 20 ng/mL IFN-γ (R&D systems, Minneapolis, MN, USA). 20 μM of BrdU were added 24 h before cell fixation. The staining procedure was the same as described above.

### 4.10. Real-Time PCR

Total RNA was extracted using TRIzol reagent (Invitrogen, Waltham, MA, USA) according to the manufacturer’s protocol. The template cDNAs were synthesized using a Superscript III RT-PCR kit (Invitrogen, Waltham, MA, USA). Then, the qPCR was performed by using TB Green^®®^ Premix Ex Taq™ II (Takara Bio. Inc., Shiga, Japan) with 1 μg of total RNA and the following primer sequences for RT-PCR: rat TGF-β (Forward: CACTCCCGTGGCTTCTAGTG; Reverse: CTGGCGAGCCTTAGTTTGGA), rat IGF2 (Forward: TCTTCTACCTGGCACTCTGCT; Reverse: ACGAACTGAAGAGCGTCCAC), rat iNOS (Forward: AGCTTCTGCCTCAAGCCATT; Reverse: TTTGTTACGGCTTCCAGCCT), rat IL-1β (Forward: TGGCAACTGTCCCTGAACTC; Reverse: CCCAAGTCAAGGGCTTGGAA), rat CXCL10 (Forward: CCTGCAAGTCTATCCTGTCCG; Reverse: CCTTCTTTGGCTCACCGCTT), rat Amigo2 (Forward: TGCCTAGAGCTGTCAAACCG; Reverse: TGTCAGTGGCACAGATGCAA), rat Serping1 (Forward: TGCTGCTAGCTGGGGATAGA; Reverse: TCCTGTGTGCTCAATGGGAC), rat CD109 (Forward: TGACAACCCCACGAGAGAGA; Reverse: GGAGCTCACTGGAATCGGAC), rat Emp1 (Forward: TCTCCACCATTGCCAACGTC; Reverse: TAGCTCAGAGAGCCGTCACA), rat CINC-2 (Forward: ACCAGCCTTCAGGGACTGT; Reverse: GGCTATGACTTCTGTCTGGGT).

### 4.11. Cytokine Array

MG d14 were co-cultured with astrocyte feeder cells in various ratios (1:1, 1:3, and 1:10), and, as a control, an MG d14 single culture and astrocyte single culture were performed. Since the cytokine array recommended serum-free media or low-serum media, the medium for all the samples was changed to serum-free DMEM/F12, and the cells were incubated overnight. Then, the medium was collected and centrifuged at 10,000 rpm for 5 min. The cytokine array (RayBiotech, Inc., Norcross, GA, USA) was performed according to the manufacturer’s protocol. Briefly, NC membranes were blocked with blocking buffer for 30 min and incubated with the samples overnight at 4 °C. The detection antibodies were added and incubated with the samples overnight at 4 °C. The membranes were incubated with 1x HRP–Streptavidin for 2 h at RT, and the signals were visualized by the exposure of an X-ray film (AGFA, Mortsel, Belgium).

### 4.12. NO Assay

The production of NO was measured by using the Griess reagent system (Promega, Madison, WI, USA) according to the manufacturer’s instructions. Microglia-conditioned media were collected at 3, 24, and 36 h after SP or cytokine treatment. Fifty microliters of the sulfanilamide solution were added to 50 μL of the NO_2_^−^ standard and 50 μL of conditioned medium; the mixture was incubated for 10 min at RT. Then, 50 μL of the NED was added, and the mixture was incubated for 10 min at RT. The OD at a wavelength of 550 nm was measured.

### 4.13. Western Blot

In order to examine the levels of apoptotic proteins of the microglia, microglia were treated with four conditions: control, 100 nM SP, 10 ng/mL TNF-α + 10 ng/mL IFN-γ, 10 ng/mL TNF-α + 10ng/mL IFN-γ + 100nM SP, and 10 ng/mL TNF-α + 10 ng/mL IFN-γ + 100 nM SP + 1 μM RP67580, for 24 h. The cells were lysed for Western blot analysis. The cell lysates were prepared using lysis buffer (Cell Signaling, Danvers, MA, USA) containing 1% SDS (Sigma, St. Louis, MO, USA) and 2 mM PMSF (Sigma, St. Louis, MO, USA). The proteins in the lysates and conditioned medium were separated by 6–10% SDS–PAGE and transferred to nitrocellulose membranes. The blots were blocked with 5% skim milk in TBS-T and then incubated with primary antibodies at 4 °C overnight: α-Tubulin (Sigma, St. Louis, MO, USA, 1:4000), Bcl-xL (Cell Signaling, Danvers, MA, USA, 1:2000), IκB (Cell Signaling, Danvers, MA, USA, 1:2000), and BAD (Cell Signaling, Danvers, MA, USA, 1:2000). The blots were then incubated with the secondary antibodies goat anti-rabbit HRP (Bio-Rad, Hercules, CA, USA, 1:5000) and goat anti-mouse HRP (Bio-Rad, Hercules, CA, USA, 1:3000) for 1 h. The ECL signal was visualized by exposing an X-ray film to the membrane (AGFA, Mortsel, Belgium).

### 4.14. ELISA

The astrocytes were incubated with the following cytokines: 100 nM SP, 10 ng/mL IL-4, 10 ng/mL IL-10, 10 ng/mL TNF-α, or 20 ng/mL IFN-γ. The conditioned medium was collected 24 h after the cytokine treatment. The CINC-1, CINC-2, and VEGF levels were measured using rat CINC-1, CINC-2, and VEGF ELISA kits (R&D Systems, Minneapolis, MN, USA). Data from three independent experiments were statistically analyzed.

### 4.15. Statistics

All the data are presented as the mean ± SEM values. Statistical analysis of all the data was carried out using the GraphPad Prism software. The unpaired, two-tailed Student’s *t*-test was used to determine statistically significant differences (* *p*-value < 0.05).

## 5. Conclusions

In this study we demonstrated that cross-talk between astrocyte and microglia is beneficial for the microglia proliferation and M2 type acquisition and A2 type polarization of astrocyte. Furthermore, we demonstrated in this study that M1/M2 and A1/A2 polarization are interconvertible in vitro under the different cytokine milieu, simulating the situation occurring in the brain injury and brain diseases. Especially GM-CSF markedly stimulated the proliferation of ex vivo-cultured microglia, which can be successfully converted to M2 type microglia in vitro by Th2 cytokines such as IL-4, anti-inflammatory cytokine IL-10, and SP as a candidate inducer of M2 microglia. This strategical improvement of M2 microglia ex vivo culture may be important for the development of M2 microglia adoptive cell therapy or study design of interplay between M1/M2 and other brain cells.

## Data Availability

The data presented in this study are available within the article.

## References

[1] Zhang S.-C. (2001). Defining glial cells during CNS development. Nat. Rev. Neurosci..

[2] Pierre W.C., Smith P.L.P., Londono I., Chemtob S., Mallard C., Lodygensky G.A. (2017). Neonatal microglia: The cornerstone of brain fate. Brain Behav. Immun..

[3] Taylor S.E., Morganti-Kossmann C., Lifshitz J., Ziebell J.M. (2014). Rod microglia: A morphological definition. PLoS ONE.

[4] Cherry J.D., Olschowka J.A., O’Banion M.K. (2014). Neuroinflammation and M2 microglia: The good, the bad, and the inflamed. J. Neuroinflamm..

[5] Ma Y., Wang J., Wang Y., Yang G.-Y. (2016). The biphasic function of microglia in ischemic stroke. Prog. Neurobiol..

[6] Hu X., Li P., Guo Y., Wang H., Leak R., Chen S., Gao Y., Chen J. (2012). Microglia/macrophage polarization dynamics reveal novel mechanism of injury expansion after focal cerebral ischemia. Stroke.

[7] Liu X., Liu J., Zhao S., Zhang H., Cai W., Cai M., Ji X., Leak R., Gao Y., Chen J. (2016). Interleukin-4 is essential for microglia/macrophage M2 polarization and long-term recovery after cerebral ischemia. Stroke.

[8] Orihuela R., McPherson C.A., Harry G.J. (2015). Microglial M1/M2 polarization and metabolic states. Br. J. Pharmacol..

[9] Jin Y., Hong H.S., Son Y. (2015). Substance P enhances mesenchymal stem cells-mediated immune modulation. Cytokine.

[10] Jiang M.H., Chung E., Chi G.F., Ahn W., Lim J.E., Hong H.S., Kim D.W., Choi H., Kim J., Son Y. (2012). Substance P induces M2-type macrophages after spinal cord injury. Neuroreport.

[11] Lim J.E., Chung E., Son Y. (2017). A neuropeptide, Substance-P, directly induces tissue-repairing M2 like macrophages by activating the PI3K/Akt/mTOR pathway even in the presence of IFNγ. Sci. Rep..

[12] Ridet J., Privat A., Malhotra S., Gage F. (1997). Reactive astrocytes: Cellular and molecular cues to biological function. Trends Neurosci..

[13] Liu Z., Chopp M. (2015). Astrocytes, therapeutic targets for neuroprotection and neurorestoration in ischemic stroke. Prog. Neurobiol..

[14] Choudhury G.R., Ding S. (2015). Reactive astrocytes and therapeutic potential in focal ischemic stroke. Neurobiol. Dis..

[15] Pekny M., Wilhelmsson U., Pekna M. (2014). The dual role of astrocyte activation and reactive gliosis. Neurosci. Lett..

[16] Liddelow S.A., Barres B.A. (2017). Reactive astrocytes: Production, function, and therapeutic potential. Immunity.

[17] Bs J.T.H., Dawson V.L., Dawson T.M. (2019). The A1 astrocyte paradigm: New avenues for pharmacological intervention in neurodegeneration. Mov. Disord..

[18] Batlle M., Ferri L., Andrade C., Ortega F.J., Vidal-Taboada J.M., Pugliese M., Mahy N., Rodríguez M.J. (2015). Astroglia-microglia cross talk during neurodegeneration in the rat hippocampus. BioMed Res. Int..

[19] Liu W., Tang Y., Feng J. (2011). Cross talk between activation of microglia and astrocytes in pathological conditions in the central nervous system. Life Sci..

[20] Kaneko Y.S., Nakashima A., Mori K., Nagatsu T., Nagatsu I., Ota A. (2009). Lipopolysaccharide extends the lifespan of mouse primary-cultured microglia. Brain Res..

[21] Fu R., Shen Q., Xu P., Luo J., Tang Y. (2014). Phagocytosis of microglia in the central nervous system diseases. Mol. Neurobiol..

[22] Okada S., Zhang H., Hatano M., Tokuhisa T. (1998). A physiologic role of Bcl-xL induced in activated macrophages. J. Immunol..

[23] Iadecola C., Anrather J. (2011). The immunology of stroke: From mechanisms to translation. Nat. Med..

[24] Navarro-Sobrino M., Rosell A., Penalba A., Ribó M., Alvarez-Sabín J., Fernández-Cadenas I., Montaner J. (2009). Role of endogenous granulocyte-macrophage colony stimulating factor following stroke and relationship to neurological outcome. Curr. Neurovasc. Res..

[25] Norden D.M., Fenn A.M., Dugan A., Godbout J.P. (2014). TGFβ produced by IL-10 redirected astrocytes attenuates microglial activation. Glia.

[26] Nicoletti F., Zaccone P., Di Marco R., Di Mauro M., Magro G., Grasso S., Mughini L., Meroni P., Garotta G. (1996). The effects of a nonimmunogenic form of murine soluble interferon-gamma receptor on the development of autoimmune diabetes in the NOD mouse. Endocrinology.

[27] Nicoletti F., Zaccone P., Di Marco R., Lunetta M., Magro G., Grasso S., Meroni P., Garotta G. (1997). Prevention of spontaneous autoimmune diabetes in diabetes-prone BB rats by prophylactic treatment with antirat interferon-gamma antibody. Endocrinology.

[28] Debray-Sachs M., Carnaud C., Boitard C., Cohen H., Gresser I., Bedossa P., Bach J.F. (1991). Prevention of diabetes in NOD mice treated with antibody to murine IFN gamma. J. Autoimmun..

[29] Preisser T.M., da Cunha V.P., Santana M.P., Pereira V.B., Cara D.C., Souza B.M., Miyoshi A. (2021). Recombinant *Lactococcus lactis* carrying IL-4 and IL-10 coding vectors protects against type 1 diabetes in NOD mice and attenuates insulitis in the STZ-induced model. J. Diabetes Res..

[30] Gérard C., Bruyns C., Marchant A., Abramowicz D., Vandenabeele P., Delvaux A., Fiers W., Goldman M., Velu T. (1993). Interleukin 10 reduces the release of tumor necrosis factor and prevents lethality in experimental endotoxemia. J. Exp. Med..

[31] Zaccone P., Phillips J., Conget I., Gomis R., Haskins K., Minty A., Bendtzen K., Cooke A., Nicoletti F. (1999). Interleukin-13 prevents autoimmune diabetes in NOD mice. Diabetes.

[32] Sánchez-Fernández A., Zandee S., Amo-Aparicio J., Charabati M., Prat A., Garlanda C., Eisenmesser E.Z., Dinarello C.A., López-Vales R. (2021). IL-37 exerts therapeutic effects in experimental autoimmune encephalomyelitis through the receptor complex IL-1R5/IL-1R8. Theranostics.

[33] Cavalli E., Mazzon E., Basile M.S., Mammana S., Pennisi M., Fagone P., Kalfin R., Martinovic V., Ivanovic J., Andabaka M. (2019). In silico and in vivo analysis of IL37 in multiple sclerosis reveals its probable homeostatic role on the clinical activity, disability, and treatment with fingolimod. Molecules.

[34] Basile M.S., Battaglia G., Bruno V., Mangano K., Fagone P., Petralia M.C., Nicoletti F., Cavalli E. (2020). The dichotomic role of macrophage migration inhibitory factor in neurodegeneration. Int. J. Mol. Sci..

[35] Petralia M.C., Battaglia G., Bruno V., Pennisi M., Mangano K., Lombardo S.D., Fagone P., Cavalli E., Saraceno A., Nicoletti F. (2020). The role of macrophage migration inhibitory factor in Alzheimer’s disease: Conventionally pathogenetic or unconventionally protective?. Molecules.

[36] Liu B., Du L., Hong J.S. (2000). Naloxone protects rat dopaminergic neurons against inflammatory damage through inhibition of microglia activation and superoxide generation. J. Pharmacol. Exp. Ther..

